# Supplementary figures and images for: Unisexual reproduction promotes competition for mating partners in the global human fungal pathogen Cryptococcus deneoformans
Source: PLoS Genet. 2019 Sep 19;15(9):e1008394. doi: 10.1371/journal.pgen.1008394 (PMC6772093; doi:10.1371/journal.pgen.1008394)

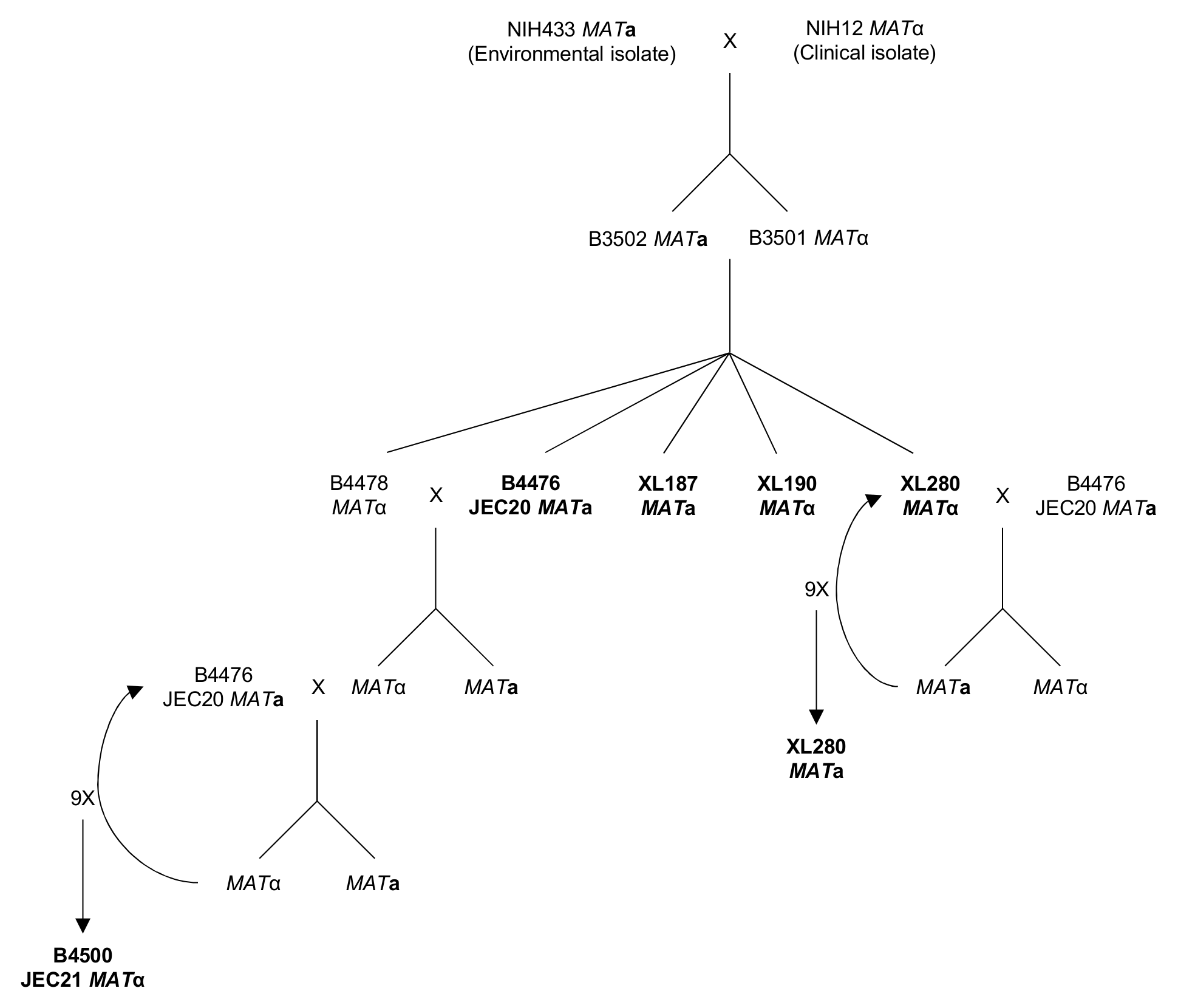

Supplement: S1 Fig — B4478 MATα, JEC20a, XL187a, XL190α, and XL280α are F2 progeny from the cross between F1 progeny B3502 MATa and B3501 MATα, which were derived from a cross between the environmental isolate NIH433 MATa and the clinical isolate NIH12 MATα. JEC20a was then crossed with B4478 MATα, and an α progeny was backcrossed with JEC20a. This process was repeated 9 times to yield the congenic partner B4500 JEC21α of B4476 JEC20a. XL280α was crossed with JEC20a, and a MATa progeny was backcrossed with XL280α. This process was repeated 9 times to yield the congenic partner XL280a. (TIF) [file pgen.1008394.s001.tif]

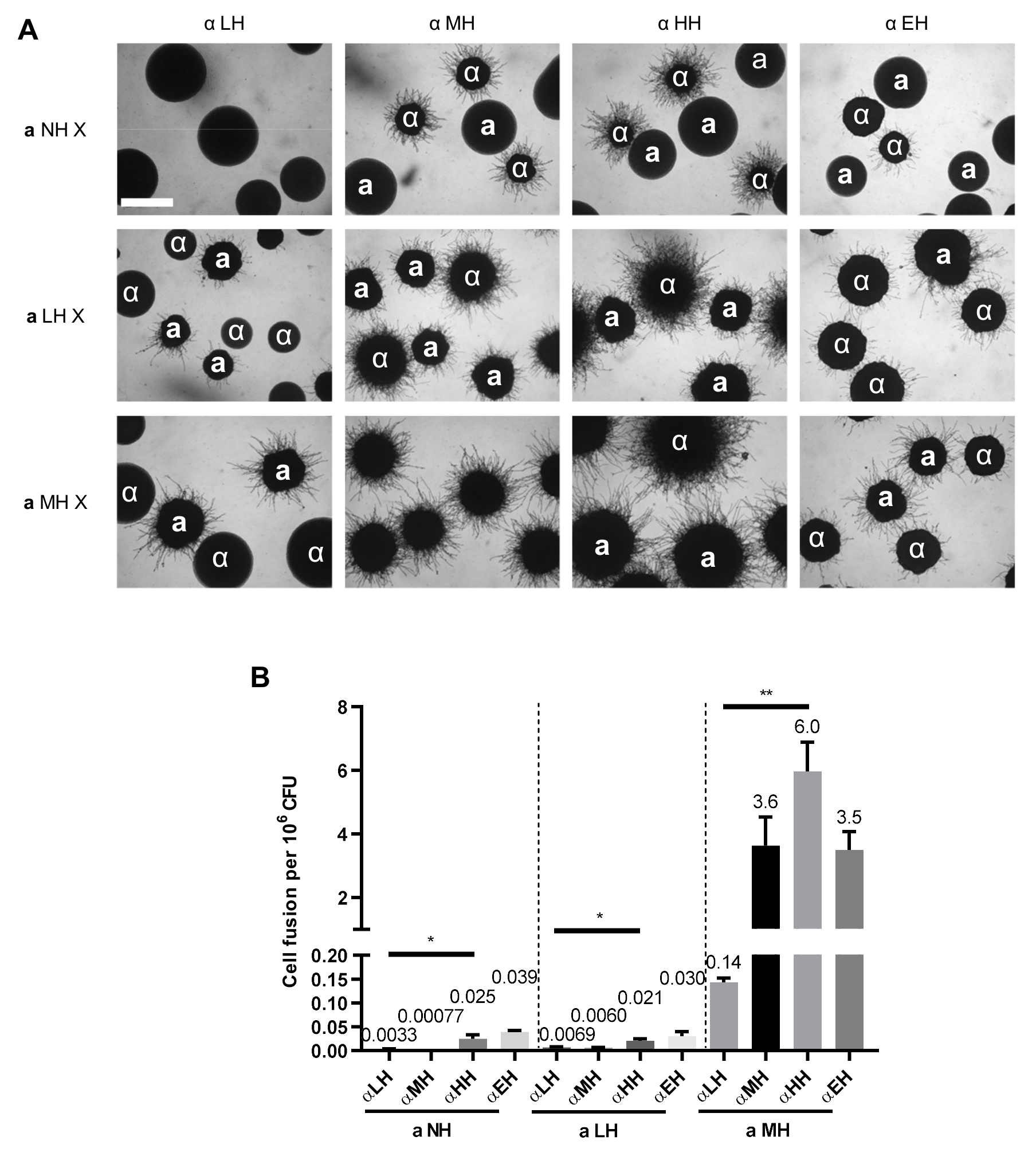

Supplement: S2 Fig — (A) LH, MH, HH, NH, and EH MATα colonies derived from single cells were grown for seven days and in some cases, hyphae facilitated contact between MATα and MATa colonies. The scale bar represents 500 μm. (B) Cell fusion frequencies between MATa and MATα mating partners for each mating pair are shown. * indicates 0.01<p≤0.05 and ** indicates 0.001<p≤0.01 for each group analysis by one-way ANOVA. (TIF) [file pgen.1008394.s002.tif]

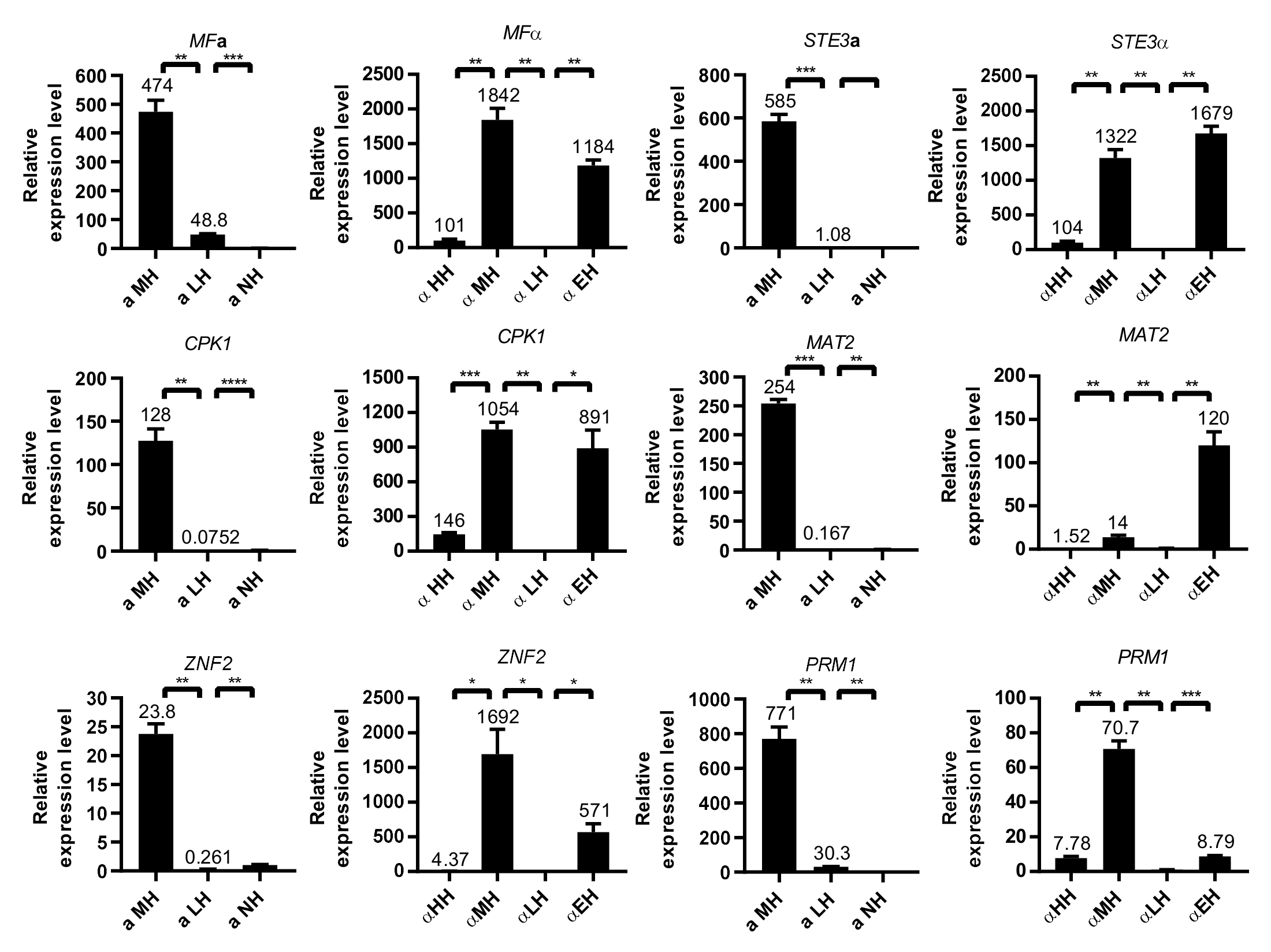

Supplement: S3 Fig — Gene expression patterns for MFα, MFa, STE3α, STE3a, CPK1, MAT2, ZNF2, and PRM1 were examined by qRT PCR (NS indicates p≤0.01, * indicates 0.01<p≤0.05, ** indicates 0.001<p≤0.01, *** indicates 0.0001<p≤0.001, and **** indicates p≤0.0001 for each pairwise comparison.). MH MATa (XL280a) and HH MATα (XL190α) strains, LH MATa (XL187a) and MHMATα (XL280α) strains, NH MATa (JEC20a) and LH MATα (JEC21α) strains, and an enhanced hyphal MATα (JEC21α gpa3Δ::NEO) strain were grown on V8 agar medium for 36 hours. The expression levels of JEC20a or JEC21α were set to 1, and the remaining values of the same mating type strains were normalized to this. The error bars represent the standard deviation of the mean for three biological replicates. (TIF) [file pgen.1008394.s003.tif]

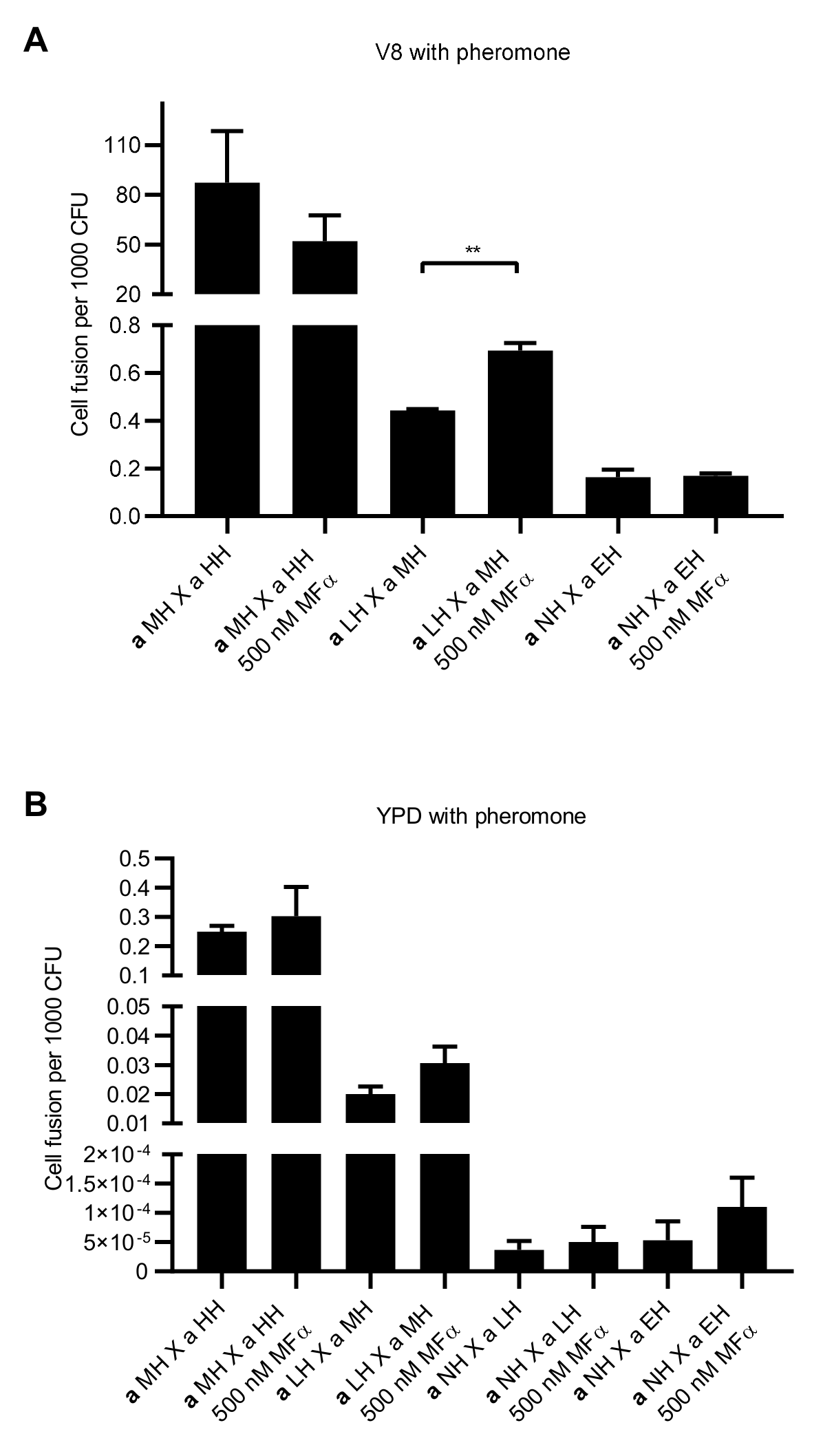

Supplement: S4 Fig — Cell fusion frequencies between MATa and MATα cells (MH and HH, LH and MH, NH and LH, and NH and EH) co-incubated on (A) V8 and (B) YPD media for four days both in the absence and in the presence of 500 nM α pheromone peptide. (TIF) [file pgen.1008394.s004.tif]

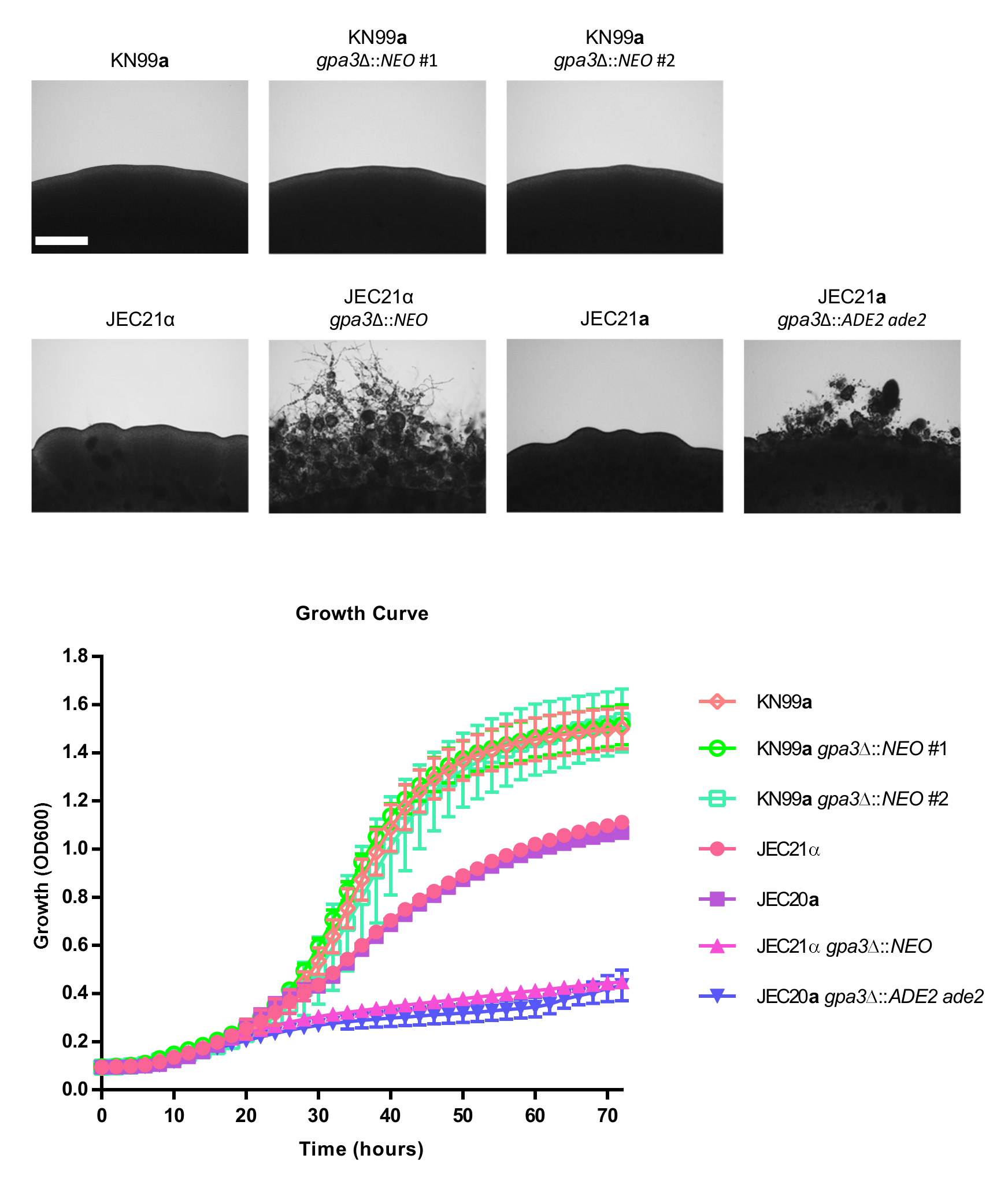

Supplement: S5 Fig — Hyphal growth on MS medium for two weeks for C. neoformans strains KN99a, KN99a gpa3Δ::NEO #1, and KN99a gpa3Δ::NEO #2, and NH, LH, and EH C. deneoformans strains JEC20a, JEC21α, JEC20a gpa3Δ::ADE2 ade2, and JEC21α gpa3Δ::NEO. The scale bar represents 500 μm. Growth curves were generated using an automated Tecan Sunrise absorbance reader bi-hourly for 72 hours. (TIF) [file pgen.1008394.s005.tif]

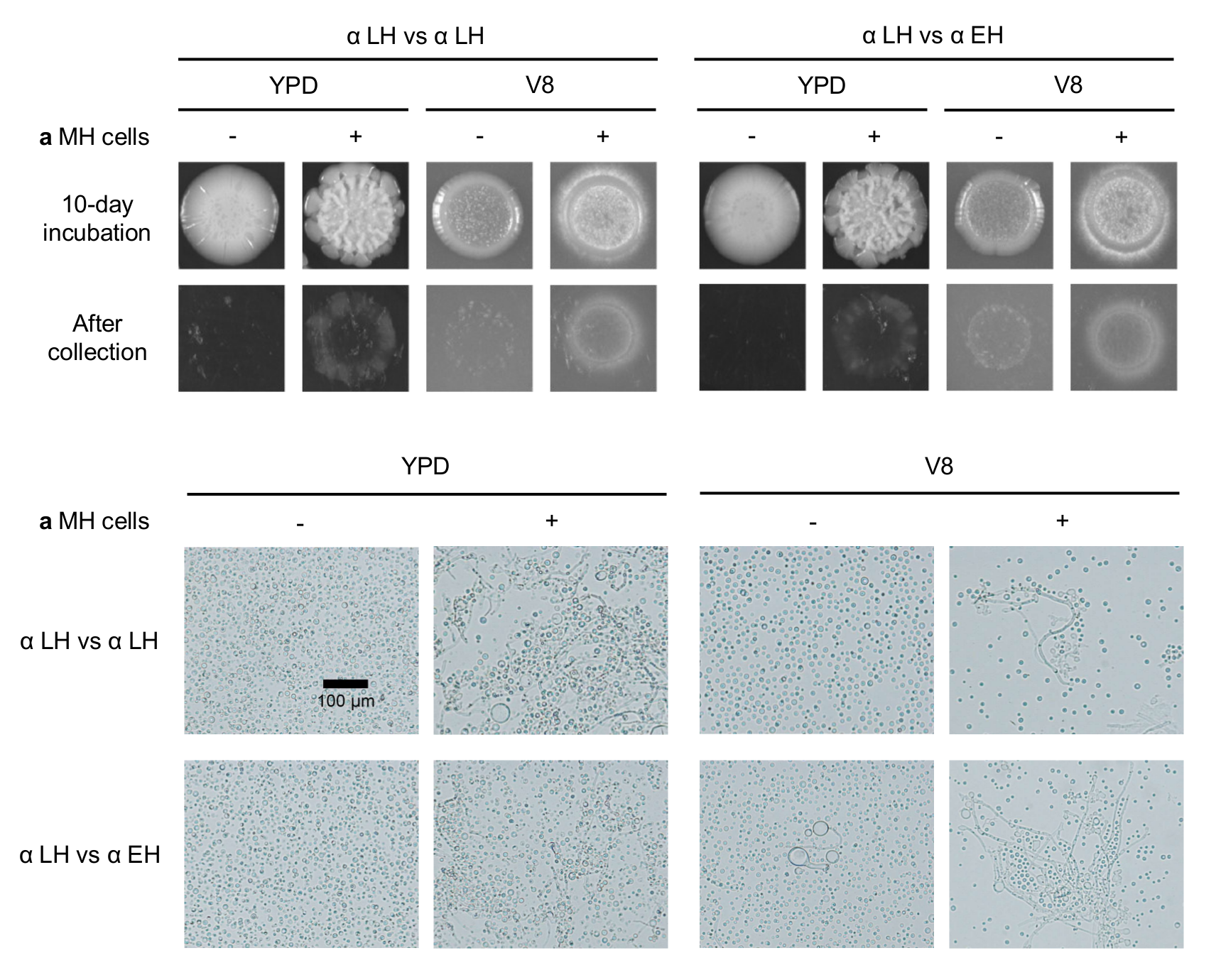

Supplement: S6 Fig — Equal number of cells of α LH and α LH strains, and of α LH and α EH strains were mixed and spot-inoculated on both YPD and V8 media both in the absence and in the presence of equal number of a MH cells. Cells were scraped off agar medium after 10 days of incubation, and both yeast cells and hyphae were collected. (TIF) [file pgen.1008394.s006.tif]
