# Supplementary material for: Unisexual reproduction promotes competition for mating partners in the global human fungal pathogen Cryptococcus deneoformans
Source: PLoS Genet. 2019 Sep 19;15(9):e1008394. doi: 10.1371/journal.pgen.1008394 (PMC6772093; doi:10.1371/journal.pgen.1008394)
Supplement: S1 Table — (DOCX) [file pgen.1008394.s007.docx]

**Table S1. Bisexual cell fusion frequencies for the mating competition experiment.**

|  | Competition pair | Fusion pair | Cell fusion per 1000 CFU | Fold difference | P value |
| --- | --- | --- | --- | --- | --- |
| Competing for **a** MH *HYG* | α-1 HH  Vs α-2 MH | α HH X **a** MH | 52 | 8.1 | 0.0005  *** |
|  |  | α MH X **a** MH | 6.4 |  |  |
|  | α-1 HH  Vs α-2 LH | α HH X **a** MH | 10 | 24 | 0.0023  ** |
|  |  | α LH X **a** MH | 0.41 |  |  |
|  | α-1 HH  Vs α-2 LH | α MH X **a** MH | 3.4 | 5.8 | 0.0159  * |
|  |  | α LH X **a** MH | 0.59 |  |  |
| Competing for **a** LH *HYG* | α-1 HH  Vs α-2 MH | α HH X **a** LH | 1.3 | 6.5 | 0.0003  *** |
|  |  | α MH X **a** LH | 0.2 |  |  |
|  | α-1 HH  Vs α-2 LH | α HH X **a** LH | 0.29 | 14.5 | 0.0228  * |
|  |  | α LH X **a** LH | 0.02 |  |  |
|  | α-1 HH  Vs α-2 LH | α MH X **a** LH | 0.091 | 4.6 | 0.0006  *** |
|  |  | α LH X **a** LH | 0.02 |  |  |
| Competing for **a** NH *HYG* | α-1 HH  Vs α-2 MH | α HH X **a** NH | 0.049 | 5.3 | 0.0468  * |
|  |  | α MH X **a** NH | 0.0092 |  |  |
|  | α-1 HH  Vs α-2 LH | α HH X **a** NH | 0.064 | 8.9 | 0.0170  * |
|  |  | α LH X **a** NH | 0.0072 |  |  |
|  | α-1 HH  Vs α-2 LH | α MH X **a** NH | 0.01 | 1.7 | 0.0126  * |
|  |  | α LH X **a** NH | 0.0059 |  |  |
