# Supplementary material for: Unisexual reproduction promotes competition for mating partners in the global human fungal pathogen Cryptococcus deneoformans
Source: PLoS Genet. 2019 Sep 19;15(9):e1008394. doi: 10.1371/journal.pgen.1008394 (PMC6772093; doi:10.1371/journal.pgen.1008394)
Supplement: S2 Table — (DOCX) [file pgen.1008394.s008.docx]

**Table S2. p-Values of one-way ANOVA analyses and Welch’s t-test for each pairwise comparison for the foraging for mating assay during mating confrontation.**

| One-way ANOVA group analyses^#^ (* 0.01<p≤0.05, ** 0.001<p≤0.01, *** 0.0001<p≤0.001, ****p≤0.0001) | | | | | | | | | | | | | |
| --- | --- | --- | --- | --- | --- | --- | --- | --- | --- | --- | --- | --- | --- |
| **a** NH X α LH | ** 0.0018 | **a** LH X α LH | * 0.0173 | **a** MH X α LH | **** <0.0001 | **a** NH X α LH | 0.0896 | **a** NH X α MH | **** <0.0001 | **a** NH X α HH | **** <0.0001 | **a** NH X α EH | **** <0.0001 |
| **a** NH X α MH |  | **a** LH X α MH |  | **a** MH X α MH |  | **a** LH X α LH |  | **a** LH X α MH |  | **a** LH X α HH |  | **a** LH X α EH |  |
| **a** NH X α HH |  | **a** LH X α HH |  | **a** MH X α HH |  | **a** MH X α LH |  | **a** MH X α MH |  | **a** MH X α HH |  | **a** MH X α EH |  |
| Pairwise Welch's t-test analyses (* 0.01<p≤0.05, ** 0.001<p≤0.01, *** 0.0001<p≤0.001, ****p≤0.0001) | | | | | | | | | | | | |  |
| **a** NH X α LH | - |  |  |  |  |  |  |  |  |  |  |  |  |
| **a** NH X α MH | * 0.0429 | - |  |  |  |  |  |  |  |  |  |  |  |
| **a** NH X α HH | * 0.0181 | * 0.0382 | - |  |  |  |  |  |  |  |  |  |  |
| **a** NH X α EH | 0.0591 | - | - | - |  |  |  |  |  |  |  |  |  |
| **a** LH X α LH | >0.9999 | - | - | - | - |  |  |  |  |  |  |  |  |
| **a** LH X α MH | - | * 0.0414 | - | - | ** 0.0076 | - |  |  |  |  |  |  |  |
| **a** LH X α HH | - | - | 0.2937 |  | 0.0507 | 0.1324 | - |  |  |  |  |  |  |
| **a** LH X α EH | - | - | - | 0.0591 | >0.9999 | - | - | - |  |  |  |  |  |
| **a** MH X α LH | 0.1594 | - | - | - | 0.1594 | - | - | - | - |  |  |  |  |
| **a** MH X α MH | - | ** 0.0078 | - | - | - | ** 0.0097 | - | - | ** 0.0077 | - |  |  |  |
| **a** MH X α HH | - | - | ** 0.0034 | - | - | - | ** 0.0032 | - | ** 0.0029 | * 0.0119 | - |  |  |
| **a** MH X α EH | - | - | - | ** 0.0011 | - | - | - | ** 0.0011 | *** 0.0010 | - | - | - |  |
|  | **a** NH X α LH | **a** NH X α MH | **a** NH X α HH | **a** NH X α EH | **a** LH X α LH | **a** LH X α MH | **a** LH X α HH | **a** LH X α EH | **a** MH X α LH | **a** MH X α MH | **a** MH X α HH | **a** MH X α EH |  |

# Group one-way ANOVA analyses were performed on three crosses between LH, MH, and HH *MAT*α cells with the same *MAT***a** cells (NH, LH, or MH), and between NH, LH, and MH *MAT***a** cells with the same *MAT*α cells (LH, MH, HH, or EH).
