# Supplementary material for: Unisexual reproduction promotes competition for mating partners in the global human fungal pathogen Cryptococcus deneoformans
Source: PLoS Genet. 2019 Sep 19;15(9):e1008394. doi: 10.1371/journal.pgen.1008394 (PMC6772093; doi:10.1371/journal.pgen.1008394)
Supplement: S3 Table — (DOCX) [file pgen.1008394.s009.docx]

**Table S3. p-Values of one-way ANOVA analyses and Welch’s t-test for each pairwise comparison for the foraging for mating assay during mating among mini-colonies.**

| One-way ANOVA group analyses^#^ (* 0.01<p≤0.05, ** 0.001<p≤0.01, *** 0.0001<p≤0.001, ****p≤0.0001) | | | | | | | | | | | | | |
| --- | --- | --- | --- | --- | --- | --- | --- | --- | --- | --- | --- | --- | --- |
| **a** NH X α LH | * 0.0269 | **a** LH X α LH | * 0.0138 | **a** MH X α LH | ** <0.0043 | **a** NH X α LH | **** <0.0001 | **a** NH X α MH | ** 0.0038 | **a** NH X α HH | *** 0.0003 | **a** NH X α EH | *** 0.0005 |
| **a** NH X α MH |  | **a** LH X α MH |  | **a** MH X α MH |  | **a** LH X α LH |  | **a** LH X α MH |  | **a** LH X α HH |  | **a** LH X α EH |  |
| **a** NH X α HH |  | **a** LH X α HH |  | **a** MH X α HH |  | **a** MH X α LH |  | **a** MH X α MH |  | **a** MH X α HH |  | **a** MH X α EH |  |
| Pairwise Welch's t-test analyses (* 0.01<p≤0.05, ** 0.001<p≤0.01, *** 0.0001<p≤0.001, ****p≤0.0001) | | | | | | | | | | | | |  |
| **a** NH X α LH |  |  |  |  |  |  |  |  |  |  |  |  |  |
| **a** NH X α MH | * 0.0341 |  |  |  |  |  |  |  |  |  |  |  |  |
| **a** NH X α HH | 0.1297 | 0.1081 |  |  |  |  |  |  |  |  |  |  |  |
| **a** NH X α EH | ** 0.0066 |  |  |  |  |  |  |  |  |  |  |  |  |
| **a** LH X α LH | * 0.0408 |  |  |  |  |  |  |  |  |  |  |  |  |
| **a** LH X α MH |  | * 0.0401 |  |  | 0.5678 |  |  |  |  |  |  |  |  |
| **a** LH X α HH |  |  | 0.6941 |  | 0.0822 | 0.071 |  |  |  |  |  |  |  |
| **a** LH X α EH |  |  |  | 0.4426 | 0.128 |  |  |  |  |  |  |  |  |
| **a** MH X α LH | ** 0.0038 |  |  |  | ** 0.0038 |  |  |  |  |  |  |  |  |
| **a** MH X α MH |  | 0.0565 |  |  |  | 0.0567 |  |  | 0.0608 |  |  |  |  |
| **a** MH X α HH |  |  | * 0.0232 |  |  |  | * 0.0231 |  | * 0.0241 | 0.1446 |  |  |  |
| **a** MH X α EH |  |  |  | * 0.0267 |  |  |  | * 0.0266 | * 0.0283 |  |  |  |  |
|  | **a** NH X α LH | **a** NH X α MH | **a** NH X α HH | **a** NH X α EH | **a** LH X α LH | **a** LH X α MH | **a** LH X α HH | **a** LH X α EH | **a M**H X α LH | **a** MH X α MH | **a** MH X α HH | **a** MH X α EH |  |

# Group one-way ANOVA analyses were performed on three crosses between LH, MH, and HH *MAT*α cells with the same *MAT***a** cells (NH, LH, or MH), and between NH, LH, and MH *MAT***a** cells with the same *MAT*α cells (LH, MH, HH, or EH).
