# Supplementary material for: Unisexual reproduction promotes competition for mating partners in the global human fungal pathogen Cryptococcus deneoformans
Source: PLoS Genet. 2019 Sep 19;15(9):e1008394. doi: 10.1371/journal.pgen.1008394 (PMC6772093; doi:10.1371/journal.pgen.1008394)
Supplement: S4 Table — (DOCX) [file pgen.1008394.s010.docx]

**Table S4. Strains and plasmids used in this study.**

|  | **Strain name** | **Genotype** | **Background** | **Sources** |
| --- | --- | --- | --- | --- |
|  | XL190α |  |  | [1] |
|  | XL280α |  |  | [1] |
|  | JEC21α |  |  | [2] |
|  | XL280**a** |  |  | [3] |
|  | XL187**a** |  |  | [4] |
|  | JEC20**a** |  |  | [2] |
| High Hyphal (HH) | XL557 | *NAT* | XL190α | Xiaorong Lin unpublished |
|  | CF914 | *NEO* | XL190α | This study |
| Intermediate Hyphal (MH) | CF978 | *HYG* | XL280**a** | This study |
|  | CF750 | *NAT* | XL280α | [5] |
|  | CF752 | *NEO* | XL280α | [5] |
|  | CF974 | *HYG* | XL280α | This study |
| Low Hyphal (LH) | CF931 | *HYG* | XL187**a** | This study |
|  | CF969 | *NAT* | JEC21α | This study |
|  | CF759 | *NEO* | JEC21α | [5] |
| No Hyphal (NH) | CF926 | *HYG* | JEC20**a** | This study |
| Enhanced Hyphal (EH) | CF1314 | *gpa3*∆::*NEO* | JEC21α | This study |
|  | CF963 | *crg1*∆::*NAT* | XL280α | This study |
|  | CF1027 | *gpa3*∆::*NEO* | XL280**a** | This study |
|  | CF1048 | *crg1*∆::*NAT gpa3*∆::*NEO* | XL280α | This study |
|  | YPH86 | *gpa3*∆::*ADE2 ade2* | JEC20**a** | [6] |
| *C. neoformans* strains | KN99**a** |  |  | [7] |
|  | YSB136 | *gpa3*∆::*NEO* | KN99**a** | [6] |
|  | YSB137 | *gpa3*∆::*NEO* | KN99**a** | [6] |
|  | **Plasmid** | **Genotype** | **Background** | **Sources** |
|  | pAI3 | *NAT AMP* |  | [8] |
|  | pJAF1 | *NEO AMP* |  | [9] |
|  | pJAF15 | *HYG AMP* |  | [9] |

1. Lin, X., Hull, C.M., and Heitman, J. (2005). Sexual reproduction between partners of the same mating type in *Cryptococcus neoformans*. Nature *434*, 1017-1021.

2. Kwon-Chung, K.J., Edman, J.C., and Wickes, B.L. (1992). Genetic association of mating types and virulence in *Cryptococcus neoformans*. Infect. Immun. *60*, 602-605.

3. Zhai, B., Zhu, P., Foyle, D., Upadhyay, S., Idnurm, A., and Lin, X. (2013). Congenic strains of the filamentous form of *Cryptococcus neoformans* for studies of fungal morphogenesis and virulence. Infect. Immun. *81*, 2626-2637.

4. Lin, X., Huang, J.C., Mitchell, T.G., and Heitman, J. (2006). Virulence attributes and hyphal growth of *C. neoformans* are quantitative traits and the *MAT*α allele enhances filamentation. PLoS Genet. *2*, e187.

5. Fu, C., and Heitman, J. (2017). *PRM1* and *KAR5* function in cell-cell fusion and karyogamy to drive distinct bisexual and unisexual cycles in the *Cryptococcus* pathogenic species complex. PLoS Genet. *13*, e1007113.

6. Hsueh, Y.P., Xue, C., and Heitman, J. (2007). G protein signaling governing cell fate decisions involves opposing Gα subunits in *Cryptococcus neoformans*. Mol. Biol. Cell *18*, 3237-3249.

7. Nielsen, K., Cox, G.M., Wang, P., Toffaletti, D.L., Perfect, J.R., and Heitman, J. (2003). Sexual cycle of *Cryptococcus neoformans* var. *grubii* and virulence of congenic **a** and α isolates. Infect. Immun. *71*, 4831-4841.

8. Idnurm, A., Reedy, J.L., Nussbaum, J.C., and Heitman, J. (2004). *Cryptococcus neoformans* virulence gene discovery through insertional mutagenesis. Eukaryot. Cell *3*, 420-429.

9. Fraser, J.A., Subaran, R.L., Nichols, C.B., and Heitman, J. (2003). Recapitulation of the sexual cycle of the primary fungal pathogen *Cryptococcus neoformans* var. *gattii*: implications for an outbreak on Vancouver Island, Canada. Eukaryot. Cell *2*, 1036-1045.
