# Supplementary material for: Unisexual reproduction promotes competition for mating partners in the global human fungal pathogen Cryptococcus deneoformans
Source: PLoS Genet. 2019 Sep 19;15(9):e1008394. doi: 10.1371/journal.pgen.1008394 (PMC6772093; doi:10.1371/journal.pgen.1008394)
Supplement: S5 Table — (DOCX) [file pgen.1008394.s011.docx]

**Table S5. Primers used in this study.**

| **Primer name** | **Sequence (5’ to 3’)** | **Description** |
| --- | --- | --- |
| M13F | GTAAAACGACGGCCAGT | *NAT*/*NEO* cassette |
| M13R | CAGGAAACAGCTATGAC | *NAT*/*NEO* cassette |
| JOHE41446 | GCCGTGCAAGGGTGTAGG | *SXI1*α F |
| JOHE41447 | GGGCCATTGGAGGAAGCTG | *SXI1*α R |
| JOHE41444 | CGGACGAGCTCTCAAATTGG | *SXI2***a** F |
| JOHE41445 | TTTGCTCGCTCTCCTTCCAC | *SXI2***a** R |
| JOHE43073 | CATTGAAACTCCCTGCTTGG | *GPA3* 5'UTR F |
| JOHE43075 | ACTGGCCGTCGTTTTACCGTCTGAAAGTTGGTCGTTG | *GPA3* 5'UTR R |
| JOHE43077 | GTCATAGCTGTTTCCTGTCTCTCTGTGGCTCGATTT | *GPA3* 3'UTR F |
| JOHE43078 | GGAACTCGCCCTCAATCTC | *GPA3 3*'UTR R |
| JOHE43072 | GCAAGAAGAGGTGAGCAGTC | *GPA3* Junction F |
| JOHE43079 | ACGTTCGTAAAGGGGTTGG | *GPA3* Junction R |
| JOHE43074 | CCGAGCATCAGACGAACAC | *GPA3* F |
| JOHE43076 | AATAGCGAGACGCACATCC | *GPA3* R |
| JOHE43065 | CGGGTTGCTTTATCTCGTTC | *CRG1* 5'UTR F |
| JOHE43067 | ACTGGCCGTCGTTTTACTTATCCCAGGCAGCGTTCT | *CRG1* 5'UTR R |
| JOHE43069 | GTCATAGCTGTTTCCTGTCGCTTCTTTCCCCGATCTAC | *CRG1* 3'UTR F |
| JOHE43070 | AGAGGCTTCGGCAAGATCAT | *CRG1 3*'UTR R |
| JOHE43064 | TTTCCCTTCTGTCCCCATC | *CRG1* Junction F |
| JOHE43071 | TCGAGATGCTGGTAGGCACA | *CRG1* Junction R |
| JOHE43066 | TCTTCTTCTCTCTCGCCTCCT | *CRG1* F |
| JOHE43068 | GAATGTCGTAGTGGTCGTGGT | *CRG1* R |
| JOHE44120 | GTCTCCACTGATTTCATTGGCTCTAC | *GPD1* RTPCR F |
| JOHE44121 | GTAACCATACTCATTGTCATACCAGCTG | *GPD1* RTPCR R |
| JOHE43005 | ATCTTCACCACCTTCACTTCT | *MF*α RTPCR F |
| JOHE43006 | CTAGGCGATGACACAAAGG | *MF*α RTPCR R |
| JOHE45716 | GGACGCCTTCACTGCTATCT | *MF***a** RTPCR F |
| JOHE45717 | GCTACCGTAAGCCTCTTCGTT | *MF***a** RTPCR R |
| JOHE44039 | CTCCTTGTCCTTTTACCTCTGC | *STE3*α RTPCR F |
| JOHE44040 | CTTGTGGCTGAAATCCCAAC | *STE3*α RTPCR R |
| JOHE45718 | GACGGTATCACTGGTTGTCT | *STE3***a** RTPCR F |
| JOHE45719 | CGTACTATCCTCGCTTCATC | *STE3***a** RTPCR R |
| JOHE44033 | CAGGTTCAACGTCGGCAACAACTA | *CPK1* RTPCR F |
| JOHE44034 | TCAAGTCGCGATGGATGATTTCAGCAG | *CPK1* RTPCR R |
| JOHE44027 | AGGAAGCTCGAGCATCGAAAGCTGTAT | *MAT2* RTPCR F |
| JOHE44028 | TGAGCTGCAGTAGCTCGTAAATCTGAC | *MAT2* RTPCR R |
| JOHE44029 | ACCCTTTCCAACCCCTTGTCAACG | *ZNF2* RTPCR F |
| JOHE44030 | AAGGACGTTTCCCAGTGTGAATACGCC | *ZNF2* RTPCR R |
| JOHE42831 | TGCCTCTTCTTCCGTATCGT | *PRM1* RTPCR F |
| JOHE42832 | CCCCAAAGGGATCTTTTCTC | *PRM1* RTPCR R |
