# Supplementary material for: Unisexual reproduction promotes competition for mating partners in the global human fungal pathogen Cryptococcus deneoformans
Source: PLoS Genet. 2019 Sep 19;15(9):e1008394. doi: 10.1371/journal.pgen.1008394 (PMC6772093; doi:10.1371/journal.pgen.1008394)
Supplement: S6 Table — (DOCX) [file pgen.1008394.s012.docx]

**Table S6. Mating competition experimental design.**

|  | Unisex | #1 α | #2 α |  |
| --- | --- | --- | --- | --- |
| #1 | HH X HH | XL190α *NAT* | XL190α *NEO* |  |
| #2 | MH X MH | XL280α *NAT* | XL280α *NEO* |  |
| #3 | LH X LH | JEC21α *NAT* | JEC21α *NEO* |  |
|  | Bisex | #1 α | #2 **a** |  |
| #4 | HH X MH | XL190α *NAT* | XL280**a** *HYG* |  |
| #5 | MH X LH | XL280α *NAT* | XL187**a** *HYG* |  |
| #6 | LH X NH | JEC21α *NAT* | JEC20**a** *HYG* |  |
|  | Unisex competition | #1 α | #2 α | #3 α |
| #7 | MH/HH/LH | XL280α *HYG* | XL190α *NAT* | JEC21α *NEO* |
|  | Bisex competition | #1 α | #2 α | #3 **a** |
| #8 | MH/HH/MH | XL280α *NAT* | XL190α *NEO* | XL280**a** *HYG* |
| #9 | MH/LH/MH | XL280α *NAT* | JEC21α *NEO* | XL280**a** *HYG* |
| #10 | HH/LH/MH | XL190α *NAT* | JEC21α *NEO* | XL280**a** *HYG* |
| #11 | MH/HH/LH | XL280α *NAT* | XL190α *NEO* | XL187**a** *HYG* |
| #12 | MH/LH/LH | XL280α *NAT* | JEC21α *NEO* | XL187**a** *HYG* |
| #13 | HH/LH/LH | XL190α *NAT* | JEC21α *NEO* | XL187**a** *HYG* |
| #14 | MH/HH/NH | XL280α *NAT* | XL190α *NEO* | JEC20**a** *HYG* |
| #15 | MH/LH/NH | XL280α *NAT* | JEC21α *NEO* | JEC20**a** *HYG* |
| #16 | HH/LH/NH | XL190α *NAT* | JEC21α *NEO* | JEC20**a** *HYG* |
|  | Unisex | #1 α | #2 α |  |
| #17 | LH/LH | JEC21α *NEO* | JEC21α *NAT* |  |
| #18 | IH/LH | JEC21α *gpa3::NEO* | JEC21α *NAT* |  |
|  | Unisex competition | #1 α | #2 α | #3 α |
| #19 | LH/LH/MH | JEC21α *NEO* | JEC21α *NAT* | XL280α *HYG* |
| #20 | IH/LH/MH | JEC21α *gpa3::NEO* | JEC21α *NAT* | XL280α *HYG* |
|  | Bisex | #1 α | #2 α |  |
| #21 | LH/NH | JEC21α *NEO* | JEC20**a** *HYG* |  |
| #22 | IH/NH | JEC21α *gpa3::NEO* | JEC20**a** *HYG* |  |
|  | Bisex competition | #1 α | #2 α | #3 **a** |
| #23 | LH/LH/NH | JEC21α *NEO* | JEC21α *NAT* | JEC20**a** *HYG* |
| #24 | IH/LH/NH | JEC21α *gpa3::NEO* | JEC21α *NAT* | JEC20**a** *HYG* |
